# Comparison of offspring outcomes in women with and without epilepsy

**DOI:** 10.1002/acn3.52316

**Published:** 2025-02-03

**Authors:** Huali Luo, Xiaomin Mao, Shuli Zhu, Qiong Luo, Jiajia Fang, Qiwei Li

**Affiliations:** ^1^ Department of Pathology The Fourth Affiliated Hospital, Zhejiang University School of Medicine Zhejiang China; ^2^ Department of Nursing The Fourth Affiliated Hospital, Zhejiang University School of Medicine Zhejiang China; ^3^ Department of Obstetrics People's Hospital of Jinhua City Zhejiang China; ^4^ Department of Obstetrics Women's Hospital, Zhejiang University School of Medicine Hangzhou China; ^5^ Department of Neurology The Fourth Affiliated Hospital, Zhejiang University School of Medicine Zhejiang China; ^6^ Department of Rehabilitation The Children's Hospital, Zhejiang University School of Medicine, National Clinical Research Center for Child Health Hangzhou China

## Abstract

**Objective:**

The potential impact of antiseizure medications (ASMs) on abortion rate and bone metabolism in the offspring of pregnant women with epilepsy (WWE) is currently unknown. This research aimed to assess the potential risk by conducting a comparative analysis of bone metabolism‐related indicators in the offspring of WWE.

**Methods:**

We retrospectively analyzed data from 83 epileptic parturients receiving antenatal care at our hospital and a co‐operative hospital from January 1, 2012, to December 31, 2021, comparing them to a control group of 249 parturients. The study analyzed and compared the two groups' growth parameters, including delivery mode, femoral length, biparietal diameter, and birth weight. Differences in femoral length, biparietal diameter, and birth weight among different ASM groups were also examined.

**Results:**

WWE were more likely to undergo a cesarean section with a lower abortion rate (55.4% vs. 37.3%, *P* = 0.004). After adjusting for potential confounding variables, offspring femoral length in WWE was significantly reduced compared to the control group (6.812 cm vs. 6.923 cm, *P* < 0.0001). Moreover, those born to WWE using multiple ASMs had significantly reduced femoral and biparietal lengths compared to those whose mothers used a single ASM or none (*P* < 0.0001). Additionally, birth weight was significantly lower in offspring of WWE using multiple ASMs than those not using ASM (*P* < 0.05).

**Interpretation:**

WWE experienced fewer abortions but worse negative offspring outcomes. The bone metabolism of the offspring of WWE was decreased and exhibited shortened femoral length, particularly in those on multiple ASMs.

## Introduction

Epilepsy is one of the most common neurological disorders, affecting approximately 7.60‰ of the global population,^1^ which is characterized by recurrent and transient central nervous system dysfunction caused by excessive neuronal discharge. Antiseizure medications (ASMs) constitute the primary treatment modality, with some individuals requiring lifetime ASM therapy for seizure control, making the management of drug therapy a critical focus of clinical practice. In particular, women with epilepsy (WWE) need to take ASMs during pregnancy to reduce the adverse effects of seizures on pregnant women and their fetuses.^2^ Nevertheless, due to decreased enzyme protein binding, altered liver metabolism, and increased renal blood flow during pregnancy, WWE may necessitate higher doses of ASMs to mitigate the elevated risk of seizures, particularly in the initial trimester.^3^ Data show that about one in 200 pregnant women suffer from epilepsy in United States.^4^ A cross‐sectional study in China reported that the prevalence of epilepsy in pregnant women was about 2.5‰.^5^ Nevertheless, given China's large population and the gradual liberalization of the two‐and three‐child policy, the management of WWE during pregnancy in China remains challenging.

However, the administration of ASMs during pregnancy can also have detrimental effects on the fetus. Past studies have demonstrated that ASM use during pregnancy leads to an increased risk of fetal growth defects and neurodevelopmental delays.^6^ Notably, the adverse effects of valproate (VPA) on pregnant women and fetuses are especially pronounced, leading to its classification as a drug to avoid during pregnancy by the US Food and Drug Administration and Chinese.^7^ On the contrary, levetiracetam (LEV) and lamotrigine (LTG) are frequently recommended as the first‐line medications for WWE during pregnancy because of their lower teratogenic risk.

Recent studies have increasingly highlighted that people with epilepsy (PWE) have significantly lower bone mineral density (BMD) and an increased risk of fracture compared to the general population. Beerhorst et al.^8^ showed that almost 80% of PWE have low BMD, with 48.2% experiencing osteopenia and 31.8% presenting with osteoporosis. It has been widely recognized that enzyme‐induced antiseizure medications (EI‐ASMs) and VPA cause a decrease in BMD in PWE.^9^ Specifically, carbamazepine (CBZ) has been associated with increased bone loss and fracture risk, alterations in bone and mineral metabolism, evaluated bone turnover, and reduced BMD.^10^ The mechanisms by which ASMs contribute to decreased BMD have been partially elucidated. For example, EI‐ASMs activate the liver enzyme system, resulting in diminished vitamin D activity, while also impairing gastrointestinal calcium absorption, enhancing skeletal calcium mobilization, and reducing bone mineralization.^11^, ^12^ Thus, Kanner et al.^13^ have emphasized the importance of monitoring osteopenia/osteoporosis when using EI‐ASMs or VPA. In addition, LEV, LTG, and lacosamide are recommended for PWE with osteopenia or osteoporosis. However, research on the impact of newer ASMs on bone health is limited and yields inconsistent findings. For example, studies have suggested that LEV affects the level of estrogen, potentially increasing fracture risk in women undergoing long‐term LEV therapy,^14^ whereas other studies have not corroborated these observations.^11^ Similarly, LTG has been reported to have adverse effects on bone development, such as bone loss, reduced BMD, and elevated markers of bone turnover,^11^ while other studies present contradictory findings.^15^

In addition, it is not clear whether the offspring of WWE are at risk of abnormal bone metabolism. Furthermore, fetal vitamin D deficiency is associated with an increased risk of fetal growth restriction, childhood asthma, and Type 1 diabetes.^16^, ^17^ Given that fetal vitamin D levels are mainly dependent on maternal levels,^18^ and considering that pregnant women, particularly in developing countries,^19^, ^20^ are at high risk for vitamin D deficiency, it is of great clinical significance to determine whether there is low bone metabolism in offspring of WWE.

In our study, we aimed to explore whether the offspring of WWE are at risk of low bone metabolism. We retrospectively analyzed the growth and developmental outcomes of WWE offspring delivered in our hospital over the past decade to determine if there is a risk of growth restriction among offspring and assess the risk of ASMS to their growth.

## Materials and Methods

In this study, we retrospectively collected all puerpera diagnosed with epilepsy from 1 January 2012 to 31 December 2021, who visited the fourth affiliated hospital, Zhejiang University School of Medicine, or the Obstetrics and Women's Hospital, Zhejiang University School of Medicine. The healthy puerpera of the same period were also taken as the control group. The procedure of this study was approved by the Ethics Committee of the aforementioned hospital (Ethics number: K2022055, IRB‐20210311‐R).

### Participants and definition

All WWE who attended either the outpatient or inpatient departments of the hospital for antenatal care from January 2012 to 31 December 2021 were subject to our study, as recorded in the electronic medical system. The inclusion criteria for the WWE were as follows: (1) age ranged from 18 to 45; (2) singleton live birth; and (3) were diagnosed with epilepsy, which met the diagnostic criteria of the revised 2010 definition of the International League Against Epilepsy.^21^ The inclusion criteria of the control group were as follows: (1) pregnant women aged 18–45 receiving antenatal care from January 2012 to 31 December 2021; and (2) singleton live births. WWE and control group were excluded if they were the following: (1) age below 18 or above 45; (2) twin or multiple pregnancies; and (3) pre‐existing conditions before pregnancy that would affect the fetal growth and development during pregnancy, like hypoparathyroidism,^22^ systemic sclerosis,^23^ celiac disease,^24^ or the use of medications affecting bone metabolism such as glucocorticoid or rifampicin. In cases where participants had multiple pregnancies meeting the above inclusion criteria, each pregnancy was considered separately. To avoid the influence of gestational weeks on the growth and developmental outcomes of offspring, a threefold control group with matched gestational weeks was included in our study.

### Data extraction

Data on the following aspects of the participants were collected from the electronic medical records or obtained through follow‐up by physicians and trained researchers: (1) Demographic characteristics of the parturients, including gestational age, ethnicity, educational level, height, pre‐pregnancy weight, and weight before delivery. Other potential confounders, such as the history of diabetes or hypertension before or during pregnancy, were also collected. (2) Epilepsy‐related characteristics, including whether to take ASMs and detailed names. (3) Offspring outcomes, including gender, gestational weeks (determined by the last menstrual period or ultrasound, with the latter being preferred in cases of a significant discrepancy), Apgar scores at 1 and 5 minutes, birth weight, biparietal diameter, and femoral length conducted by ultrasound. Routine fetal ultrasound testing is recommended during the second and third trimesters to evaluate fetal growth and development. Considering the stability of the data, we included ultrasound data from the third trimester (≥28 weeks) for statistical analysis. Besides, we also calculated the proportion of small for gestational age and weight below 2500 g based on a semi‐customized fetal growth curve based on the Chinese population.^25^

### Statical analysis

The statistical analysis was conducted using SPSS version 26.0 (Statistical Package for Social Sciences, IBM). The mean ± standard deviation was used to report quantitative data, whereas percentages were employed to present categorical variables. Chi‐squared test was conducted to compare the differences in categorical variables (such as nationality and education level) between the two groups, *t*‐test was conducted to compare the differences in continuous variables (such as age and height) between the two groups, while one‐way analysis of variance was conducted to compare continuous variables (such as birth weight) among the three groups. Covariance analysis was used to eliminate the confounding factors in the measurement of fetal birth weight, biparietal diameter, and femoral length. Major confounders included the maternal age at delivery, height, pre‐pregnancy weight and body mass index (BMI), pre‐delivery weight and BMI, gestational weeks (gestational week at birth and gestational week measured by ultrasound), and history of drinking and smoking during pregnancy.

## Results

### Comparison of clinical characteristics

A total of 83 pregnant WWE and 249 healthy controls were included in our study. Compared to the control group, WWE had a lower education (*P* = 0.025) and a reduced incidence of abortion (*P* = 0.022). No significant differences were observed between the two groups in terms of age, ethnicity, history of smoking and drinking during pregnancy, height, pre‐pregnancy weight and BMI, pre‐delivery weight and BMI, weight gain during pregnancy, or the presence of pre‐pregnancy or gestational diabetes mellitus, and hypertension. Detailed figures are shown in Table 1.

**Table 1:** Comparison of clinical characteristics between the control group and WWE.

Clinical characteristics	Control group (*N* = 249)	WWE (*N* = 83)	*P* value
Age (Year)	29.5 ± 4.3	29.0 ± 3.7	0.400
Ethnicity (%)			
Han	99.6	98.8	NA
other	0.4	1.2	
Education level (%)			
Below junior high	16.9	26.5	0.025*
Senior high	22.5	10.8	
Upon senior high	60.6	62.7	
History of smoking (%)			
Yes	0	0	NA
No	100	100	
History of alcohol (%)			
Yes	0	0	NA
No	100	100	
Pre‐pregnancy weight (Kg)	55.4 ± 8.1	53.9 ± 9.0	0.158
Pre‐delivery weight (Kg)	68.6 ± 8.3	66.9 ± 9.8	0.119
Weight gain (Kg)	13.3 ± 3.9	13.0 ± 4.8	0.589
Pre‐pregnancy BMI	21.6 ± 3.4	21.0 ± 3.1	0.129
Pre‐delivery BMI	26.7 ± 3.6	26.0 ± 3.2	0.104
History of diabetes			
Yes	15.3	17.1	0.727
No	84.7	82.9	
History of hypertension			
Yes	4.8	8.5	0.220
No	95.2	91.5	
Number of abortion			
0	50.6	65.1	0.022*
≥1	49.4	34.9	

*p < 0.05.

### Causes of abortion in WWE

**Figure 1:**
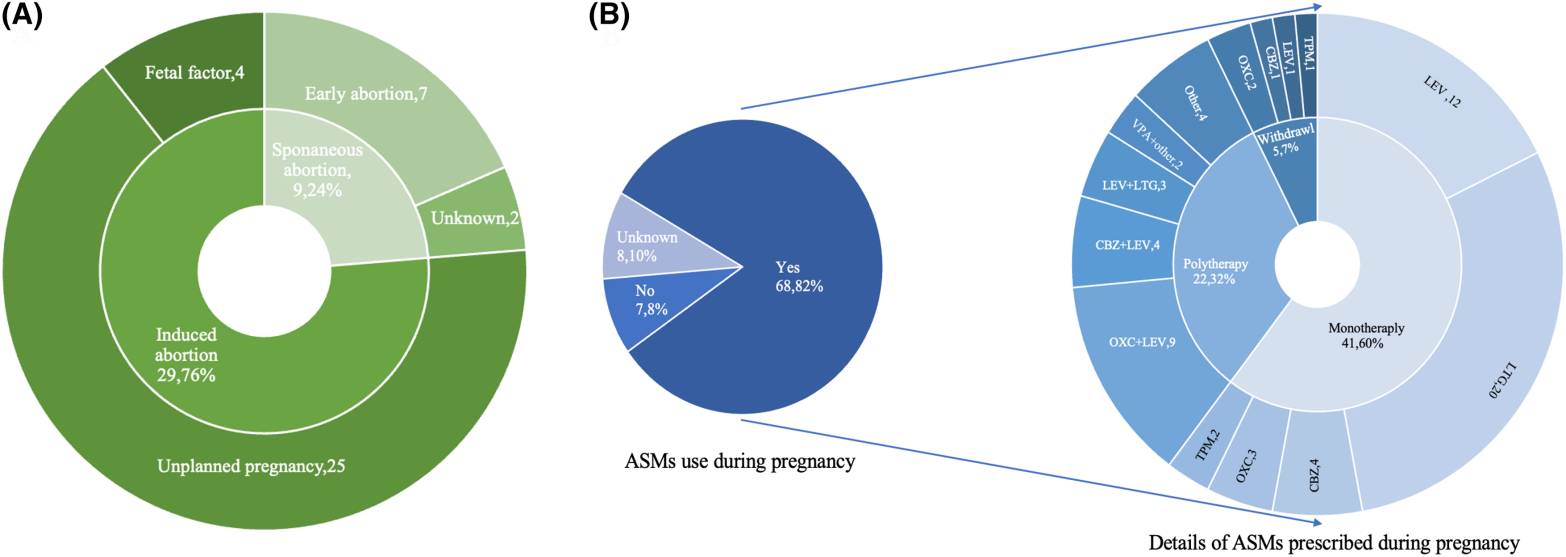
(A) Causes of abortion in WWE. (B) ASMs prescribed in WWE during pregnancy, the little circle on the left indicates the ASMs used in WWE during pregnancy, while the large circle on the right indicates details of ASMs prescribed in those WWE who use ASMs during pregnancy. ASM, antiseizure medications; CBZ, carbamazepine; LEV, levetiracetam; LTG, lamotrigine; OXC, oxcarbazepine; TPM, topiramate; VPA, valproate; WWE, women with epilepsy.

Of the 83 WWE patients included in our study, 38 (38/137) had previously experienced either induced or spontaneous abortions. Telephone follow‐up revealed that 25 WWE opted for abortions due to unintended pregnancies. There were seven (7/38) cases of early spontaneous abortions and four cases prompted by fetal factors, with some lost to follow‐up, as depicted in Figure 1A.

### ASMs prescribed in WWE

Out of the 83 WWE included in our study, 68 used ASMs during pregnancy, seven did not use ASMs, and the ASM usage use was unknown for eight individuals. Among the 68 who used ASMs during pregnancy, 5 discontinued ASMs spontaneously. The majority of participants (60.3%) used a single ASM, while 32.4% employed two or more ASMs for seizure control, with LEV and LTG being the most commonly used ASMs during pregnancy. Notably, 2 WWE took VPA during pregnancy, always in combination with other ASMs. The details are presented in Figure 1B.

### Comparison of offspring outcomes

**Figure 2:**
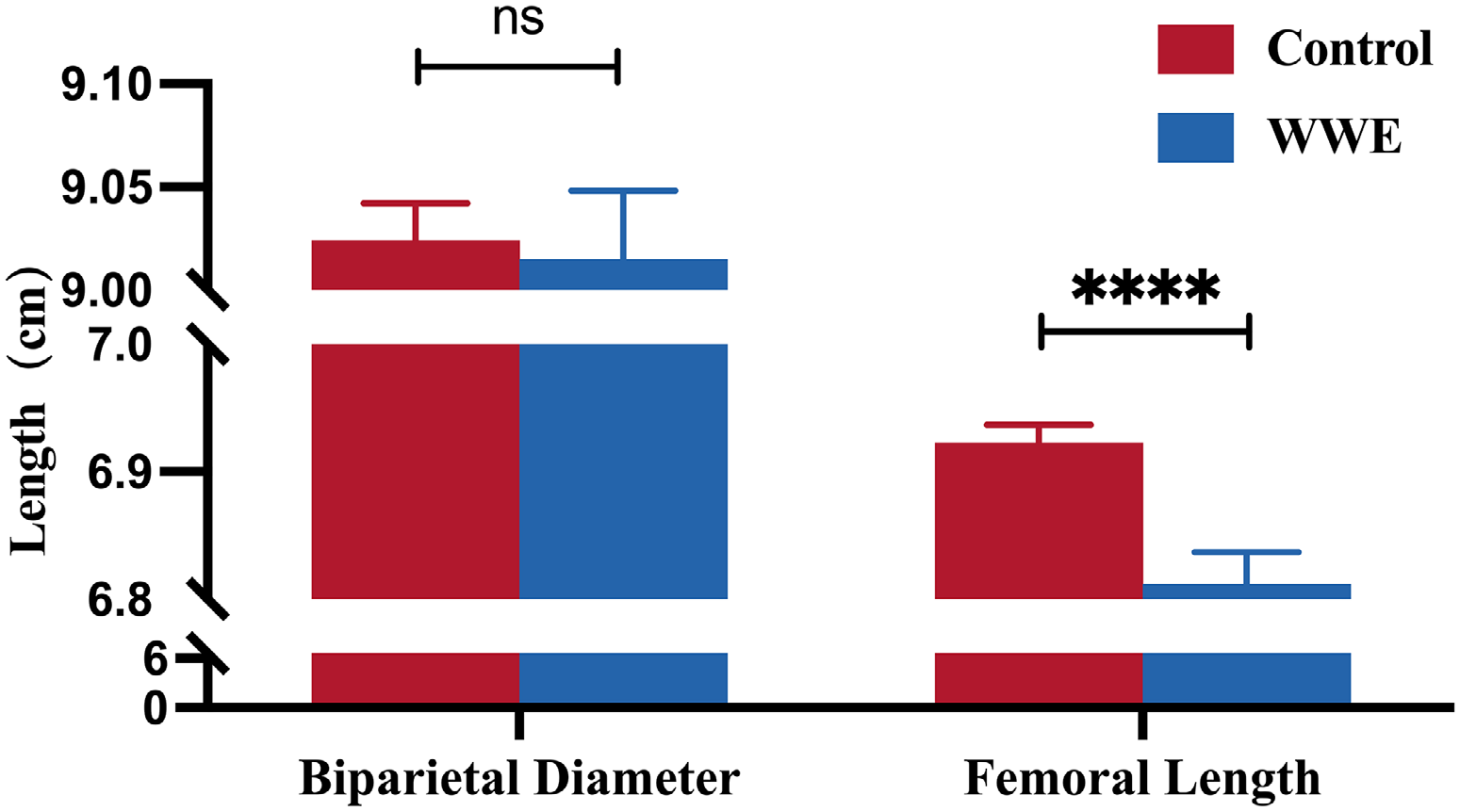
Bone length conducted by ultrasound of the control and WWE groups. ****P < 0.0001. WWE, women with epilepsy.

Compared to the control group, WWE were more likely to undergo cesarean sections (*P* = 0.004). Ultrasound measurements indicated that the femoral length was significantly shorter in the WWE group compared to the control group (Fig. 2, *P* < 0.0001). There were no significant differences between the two groups in terms of Apgar scores at 1 and 5 min, fetal birth weight, fetal sex, biparietal diameter, and the proportions of SGA and weight <2500 g, as shown in Table 2.

**Table 2:** Comparison of offspring outcomes between the control group and WWE.

Offspring outcomes	Control group (*N* = 249)	WWE (*N* = 83)	*P* value
Delivery mode(%)			
Natural childbirth	62.7	44.6	0.004**
Cesarean section	37.3	55.4	
Gender (%)			
Male	54.2	59.0	0.444
Female	45.8	41.0	
Apgar1 (%)			
≤7	2.4	2.4	NA
8‐10	97.6	97.6	
Apgar5 (%)			
≤7	0.8	0	NA
8–10	99.2	100	
Small for gestational age infant (%)			
Yes	6.4	4.8	0.594
No	93.6	95.2	
Weight < 2500 g			
Yes	10.7	8.4	0.744
No	89.3	91.6	
Birth weight^a^ (g)	3149.0 ± 23.2	3143.7.5 ± 41.3	0.911
Biparietal diameter^b^ (cm)	9.024 ± 0.018	9.015 ± 0.033	0.811
Femoral length^b^ (cm)	6.923 ± 0.014	6.812 ± 0.025	<0.0001****

**p < 0.01; ****p < 0.0001; NA: not available.

^a^ Maternal age at delivery, height, pre‐pregnancy weight, pre‐delivery weight, and gestational age at birth were used as confounders for the covariance analysis.

^b^ Maternal age at delivery, height, pre‐pregnancy weight, pre‐delivery weight, and gestational age at ultrasound examination were used as confounders for the covariance analysis.

**Figure 3:**
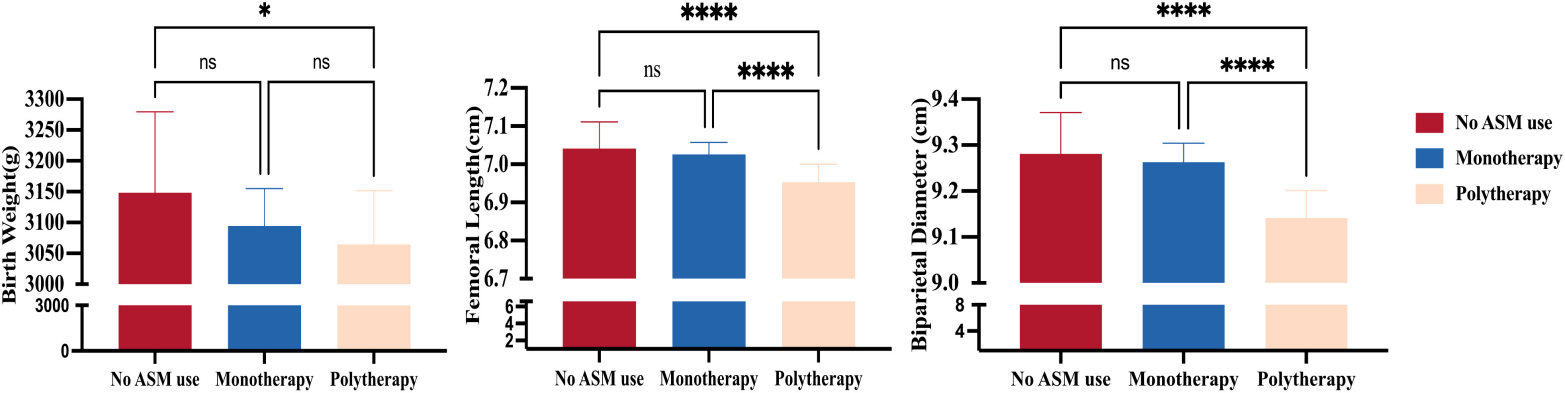
Offspring assessment indicators of the no ASM use group, monotherapy group, and polytherapy group. *P < 0.05, ****P < 0.0001. ASM, antiseizure medications.

In addition, this study compared the offspring growth among different ASMs‐prescribed groups. The assessment indicators included birth weight, femoral length, and biparietal diameter. The findings revealed that offspring in the multiple ASMs‐prescribed group had significantly lower birth weight compared to those in the non‐ASMs group (Fig. 3, *P* < 0.05). Specifically, there were significant differences in femoral length (Fig. 3, *P* < 0.0001) and biparietal diameter (Fig. 3, *P* < 0.0001) compared to those in the non‐ASMs group and the monotherapy group. However, no significant differences were found in growth parameters between the single ASM group and the non‐ASM group.

## Discussion

In this study, we retrospectively collected and compared the clinical data and birth outcomes of WWE and their offspring with those of a control group. The main findings were as follows: (1) WWE had a lower educational attainment. (2) The rate of induced abortion due to fetal abnormalities was still high among WWE compared to general Chinese women without unplanned pregnancies. Additionally, these women were more likely to undergo cesarean sections. (3) Most WWE used a single ASM to control their seizures during pregnancy, with LEV and LTG being the most commonly used. However, two women continued to use VPA during pregnancy. (4) The offspring of epileptic mothers exhibited shorter femoral length compared to the control group. (5) Offspring of mothers in the multiple ASMs group had significantly lower femoral length and biparietal diameter compared to those in the single ASMs and non‐ASMs groups. Additionally, the birth weight of offspring in the multiple ASMs group was lower than that in the non‐ASMs group.

Firstly, our study indicated that, demographically, WWE tend to have a lower level of education (*P* = 0.025). The cognitive impact of seizures,^26^ coupled with the stigma surrounding epilepsy^27^ and its limited social acceptance, may restrict educational opportunities for these patients. Therefore, it is crucial to improve epilepsy management and raise public awareness about the condition.

It should be noted that there was a significant difference in the history of abortion between pregnant WWE and the control group. Pregnant WWE exhibited an overall lower rate of abortion than controls, which is inconsistent with previous studies. Previous meta‐analyses^28^ have suggested that WWE had a higher risk of abortion compared with women without epilepsy (12 articles, 25478 pregnant women, odds ratio, OR 1.62; 95% confidence interval, CI 1.15–2.29), while some other prospective studies indicated that there were no differences in spontaneous abortion rates between WWE and control groups.^29^, ^30^ One possible explanation for our findings is the inability to differentiate between induced and spontaneous abortions within our study groups, whereas epidemiological research in China^31^ has indicated that approximately 30% of women have experienced induced abortions. Furthermore, the proportion of all types of abortion (27.7%) among pregnant WWE in the present study was notably higher than the 11.5% of abortion among WWE reported in other literature.^32^ However, when excluding abortions due to unplanned pregnancies, the abortion rate among WWE in our study aligns with existing literature.^32^ A contributing factor of high elective abortion might be that WWE are often informed that they have lower fertility compared to the general population,^33^, ^34^ which may lead them to underutilize effective contraception. Despite this perception, studies have increasingly shown no significant difference in fertility rates between WWE and the general population.^29^, ^35^ This discrepancy highlights the need for better education and support regarding contraception for WWE. Nevertheless, 13.8% (4 out of 29) underwent induced abortion due to fetal factors because of embryo arrest in our study. The incidence of abortion due to fetal factors remained higher in WWE compared to the general Chinese women, where 10% underwent abortion without an unplanned pregnancy.^36^ And seven out of nine underwent spontaneous abortion in early pregnancy, which is mainly due to fetal factors. This suggests that the risk of malformations in the offspring of WWE may still be elevated compared to the general population.^6^ Consequently, prenatal counseling and effective contraception remain crucial for WWE of childbearing age.

This study also compared delivery methods between WWE and the control group. It found that more WWE preferred cesarean sections, while the control group predominantly chose vaginal delivery (*P* = 0.004). Although existing literature suggests a higher risk of cesarean sections in WWE compared to non‐epileptic pregnant women^37^ (adjusted OR, 1.40 [95% CI, 1.38–1.42]), the Chinese guidelines published in 2021^7^ for managing epilepsy during the perinatal period recommended that most WWE can safely undergo vaginal delivery without the need for cesarean sections or early delivery. Concerns among WWE that spontaneous labor might trigger seizures are largely unfounded, given that generalized tonic–clonic seizures occur in only 1%–2% of births.^4^ To better address these concerns, it is recommended to enhance pregnancy management for WWE and to strengthen the collaboration between epilepsy specialists and obstetricians.

Our study involved 83 WWE on ASMs, with 81.9% continuing their ASMs during pregnancy. Most of these women manage their seizures with a single ASM. Notably, five women discontinued ASMs during pregnancy, while four ceased use within the first 6 months before pregnancy. Due to the inherent limitations of this retrospective study, it remains uncertain whether these WWE were well‐controlled or if they discontinued the medication on their own. Previous surveys^35^ of WWE of childbearing age have shown that many lack awareness regarding the appropriate adjustment of ASM dosages during pregnancy^29^, ^38^ and are unfamiliar with the potential risks of abrupt discontinuation.^4^ Therefore, future efforts should focus on educating WWE of childbearing age, particularly those who are pregnant, on safely managing ASMs during pregnancy. LEV and LTG remained the most commonly used ASMs during pregnancy among all WWE, consistent with previous studies^39^ identifying them as the least teratogenic options. However, two pregnant women in the study continued using VPA during pregnancy. The underlying causes for these observations are likely multifaceted, potentially encompassing inadequate pre‐pregnancy counseling, insufficient collaborative management between neurologists and obstetricians during pregnancy, unplanned pregnancies, and difficulty controlling seizures with alternative ASMs. Although no severe congenital malformations were observed in their offspring, the severe teratogenicity and other adverse effects of VPA on both mother and child are well‐documented.^7^, ^39^ As a result, minimizing the use of VPA during pregnancy remains crucial. This underscores the importance of prenatal counseling and strengthening collaborative management between epilepsy specialists and obstetricians during pregnancy.

The femoral length of the offspring of WWE was significantly shorter than that of the control group (*P* < 0.001). Although their Biparietal diameter and birthweight did not differ significantly, they exhibited a downward trend compared to the control group. A previous retrospective study^40^ that compared the effects of maternal epilepsy and ASMs on fetal growth in 164 offspring of WWE and 185 controls found evidence of fetal growth restriction and smaller head circumference in the offspring of WWE, although no difference in birth length was observed. Phenobarbital was identified as the primary ASM affecting fetal growth in that study. Similarly, a prospective investigation^41^ of 315 offspring of WWE, compared to the Italian native fetal growth curve, showed that birth weight and head circumference below the 10th percentile were observed in 15.7% and 19.2% of cases, respectively. In contrast, birth length below the 10th percentile was observed in only 1.1% of cases. VPA and phenytoin were the main ASMs associated with restricted fetal growth. However, it is important to note that both of these studies date back to around 1990. Considering that the selection of ASMs for WWE has significantly evolved with the introduction of second‐generation and newer ASMs, the impact of these newer medications on offspring growth remains to be fully understood.

In contrast, the results of this study suggest that ASMs, including newer ASMs, may still have an ongoing potential impact on the bone health of offspring born to WWE. In our study, we compared the growth parameters of the offspring of different ASM groups and found that the offspring exposed to multiple ASMs had significantly lower birth weight, femoral length, and biparietal diameter than those of the non‐ASM group. Conversely, there were no differences between the non‐ASM group and monotherapy group regarding birth weight, femoral length, and biparietal diameter. Therefore, the application of a single ASM during pregnancy is the optimal choice for pregnant WWE and their offspring, as it can not only control the seizures to reduce the harm caused by seizures during pregnancy but also minimize the restriction of ASMs on the growth and development of offspring.

Recent studies^9^ have shown that epilepsy, EIASMs, and non‐EIASMs all play an independent role in the promotion of osteoporosis. But when it comes to specific ASMs, such as the most commonly used LEV and LTG in WWE, the results are inconsistent. A small controlled study^42^ found that LEV, whether used alone or in combination, had adverse effects on bone metabolism, whereas LTG showed no such impact. Another controlled study reported no adverse effects on bone metabolism from LEV and LTG monotherapy. Conversely, other studies^43^ have suggested that LTG could have detrimental effects on bone health, including bone loss, childhood growth disorders, impaired BMD, and elevated markers of bone turnover.

However, research on bone metabolism in WWE, particularly during pregnancy and in their offspring, remains limited. Notably, previous studies^44^ have shown that vitamin D supplementation during pregnancy in healthy women significantly increases fetal femoral length and raises fetal umbilical cord blood vitamin D levels compared to controls. Some studies^45^ have also indicated a positive correlation between vitamin D levels and bone growth. Therefore, low vitamin D levels in offspring may contribute to reduced growth and development. Previous studies have shown that the vitamin D content of epileptic patients after ASM intervention was lower than that before ASM intervention. Besides, compared to monotherapy, polytherapy is associated with a high risk of abnormalities in bone mineral metabolism and vitamin D deficiency. This corroborates our findings, which observed a reduction in femoral length in the offspring of WWE, particularly in those exposed to multiple ASMs. ASMs‐prescribed may be partially responsible for vitamin D deficiency in the offspring. However, due to the retrospective nature of our study, we were unable to ascertain specific vitamin D content in the fetus, future studies are needed to investigate the relationship between growth parameters and vitamin D content in pregnant women and their fetuses.

This study has several limitations. First, as a retrospective study, it was constrained by the availability of data, with certain clinical information sent, such as seizure frequency and type^46^ in WWE, which could influence bone metabolism in epileptic patients. Second, measurements of femoral length and biparietal diameter by ultrasound are inherently subjective. Additionally, the study's sample size was relatively small and derived from only two hospitals, limiting the generalizability of the results.

## Conclusion

The reduction in bone metabolism in the offspring of pregnant WWE is evidenced by shorter femoral length, which warrants attention from clinicians. Specifically, ASMs play a significant role in the reduction of bone metabolism. Future prospective studies and investigations into bone turnover markers are needed to elucidate the effects of specific ASMs and their underlying mechanisms of action on bone health in this population.

## Author Contributions

HLL and XMM: conception and design of the study, acquisition, and analysis of data, drafting the manuscript and figures; SLZ: data acquisition; QL: manuscript review and revision; JJF: conception and design of the study, critically revised the report. QWL: conception and design of the study, critically revised the report, and accepted full responsibility for the overall content. All authors contributed to the article and approved the submitted version.

## Funding Information

This study was funded by Major science and technology projects of Zhejiang province (2023C03080) and Key projects of major health science and technology plan of Zhejiang Province (WKJ‐ZJ‐2129).

## Conflict of Interest

The authors declare no competing interests in this work.

## Data Availability

The data supporting this study's findings are available from the corresponding author upon reasonable request.
